# Interobserver reliability for manual analysis of sidestream darkfield videomicroscopy clips after resizing in ImageJ

**DOI:** 10.1186/s40635-023-00572-w

**Published:** 2023-12-08

**Authors:** Raushan C. B. Lala, Ryan A. P. Homes, Jeffrey Lipman, Mark J. Midwinter

**Affiliations:** 1 Faculty of Medicine, The University of Queensland, St Lucia, Australia; 2 Traumatic Injury Sciences Group, The University of Queensland, St Lucia, Australia; 3 Jamieson Trauma Institute, Royal Brisbane and Women’s Hospital, Herston, Australia; 4 Nimes University Hospital, University of Montpellier, Montpellier, France

**Keywords:** Critical care, Microcirculation, Interobserver, Sidestream darkfield videomicroscopy

## Abstract

**Background:**

Direct assessment of microcirculatory function remains a critical care research tool but approaches for analysis of microcirculatory videomicroscopy clips are shifting from manual to automated algorithms, with a view to clinical application in the intensive care unit. Automated analysis software associated with current sidestream darkfield videomicroscopy systems is demonstrably unreliable; therefore, semi-automated analysis of captured clips should be undertaken in older generations of software. We present a method for capture of microcirculatory clips using current version videomicroscope hardware and resizing of clips to allow compatibility with legacy analysis software. The interobserver reliability of this novel approach is examined, in addition to a comparison of this approach with the current generation of automated analysis software.

**Results:**

Resizing microcirculatory clips did not significantly change image quality. Assessment of bias between observers for manual analysis of resized clips; and between manually analysed clips and automated software analysis was undertaken by Bland–Altman analysis. Bias was demonstrated for all parameters for manual analysis of resized clips (total vessel density = 6.8, perfused vessel density = 6.3, proportion of perfused vessels = − 8.79, microvascular flow index = − 0.08). Marked bias between manual analysis and automated analysis was also evident (total vessel density = 16.6, perfused vessel density = 16.0, proportion of perfused vessels = 1.8). The difference between manual and automated analysis was linearly related to the magnitude of the measured parameter.

**Conclusions:**

Poor reliability of automated analysis is a significant hurdle for clinical translation of microcirculatory monitoring. The method presented here allows capture of microcirculatory clips using current hardware that are backwards compatible with older versions of manual analysis software. We conclude that this approach is appropriate for research applications in the intensive care unit, however the time delay to results limits utility for clinical translation.

## Background

The microcirculation is a complex network of capillaries, arterioles and venules. It is the site of gas and nutrient exchange between the blood and tissue; and hence is critical for maintenance of tissue perfusion [1]. Microcirculatory functional assessment is therefore of high research interest in intensive care cohorts. As discussed in a recent review, reliable analysis techniques are essential in establishing microcirculatory assessment as a clinical tool in the intensive care unit [2].

Microcirculatory function can be assessed by techniques that visualise the microvessels or by examination of oxygenation in a tissue block [3]. Handheld videomicroscopy (HVM) is a commonly used approach for direct visualisation of the microcirculatory network. Handheld videomicroscope technology for direct visualisation of the microcirculation has undergone rapid evolution in recent years. Orthogonal polarisation spectral imaging (commercially known as Cytoscan) was the first tool developed for direct visualisation [4]. This technique allowed imaging of the microcirculation on surfaces such as skin and mucosa.

Second generation cameras (Microscan, Microvision Medical and Capiscope Handheld Video Capillaroscopy System, KK Technology) utilise sidestream darkfield (SDF) technology to image the microcirculation. SDF involves a ring of light emitting diodes (LEDs) that apply a 540 nm wavelength to the tissue [5]. This wavelength is absorbed by haemoglobin and allows discrimination of red blood cells (containing haemoglobin) and surrounding structures. The resultant image shows the red blood cells as dark structures in visible contrast to the lighter coloured background [6]. SDF videomicrosopy has been used in many key studies examining microcirculatory function in critical illness [7, 8].

Incident darkfield (IDF) imaging is the latest generation of HVM technology. The technology is similar to SDF, however, has been optimised in several regards to improve image quality. Wavelengths are applied to the tissue in short (2 ms pulses) that are synchronised with the image acquisition to improve depth of tissue penetration and contrast between the microcirculation and surrounding structures. IDF videomicroscopes (Cytocam, Braedius) have an improved camera (14.6 megapixels) and record a larger visual field than SDF [5].

Comparisons between clips captured using SDF and IDF have been previously reported. Though both technologies produce images that are suitable for analysis, those captured using IDF are reportedly higher quality than SDF [9]. Furthermore, IDF clips had a higher vessel density than SDF clips captured in the same healthy volunteers [10]. The higher vessel density is likely related to the improved contrast, and thus vessel detection, with IDF technology.

Incident darkfield imaging, therefore, produces higher quality images and has improved sensitivity for vessel detection. It remains unclear whether the difference between SDF and IDF is clinically significant, and, whether improved sensitivity in IDF extends beyond detection of the vessel to changes in perfusion of detected vessels.

Direct visualisation techniques offer benefit over tissue block oxygenation testing in that both conductive and diffusive capacity may be assessed. These features are formally assessed by parameters derived from SDF clips and include a vascular density metric (total vessel density [TVD]) and red cell flow metrics (perfused vessel density [PVD], proportion of perfused vessels [PPV] and microvascular flow index [MFI]). The degree of heterogeneity (heterogeneity index [HI]) in the perfusion across the microvascular bed is also considered as a key parameter of microcirculatory function [11].

To ensure that clips of appropriate quality are analysed, Massey et al. [12] developed the Microcirculation Image Quality Score (MIQS). Clips are appraised prior to analysis using the MIQS to ensure that they are of appropriate length and are devoid of artefacts that compromise the validity of the generated functional parameters [12].

Traditional analysis protocols for SDF clips involve manual tracing of vessels and semi-quantitative rating of red cell flow in each identified vessel. This analysis is undertaken using Automated Vascular Analysis (AVA) version 3.2 software. Analysis of a single clip takes approximately 20 min resulting in at least a one-hour delay between image capture and result (since three clips are required for analysis). This time commitment represents a significant hurdle for the clinical use of microcirculatory assessment. Several groups (research and commercial) have therefore developed software that automates this process. Recently, the manufacturers of the Microscan SDF videomicroscope (Micro Vision Medical, Amsterdam, the Netherlands) released an updated version of AVA (4.3C) that automatically segments vessels in a clip and generates the relevant parameters.

Several studies have demonstrated a lack of agreement between data generated in AVA 4.3C (automated analysis) and AVA 3.2 (manual analysis). Guay et al. [13] compared results generated by automatic and manual analysis for clips taken in a population of surgical patients. Significant bias and non-systematic variability were demonstrated between two analysis methods. This work also assessed the ability of AVA 4.1 to discriminate between anaesthetised and unanaesthetised patients. Vessel density parameters increased significantly following induction of anaesthesia when clips were manually analysed, however no difference in any parameter was observed with automated analysis [13]. Poor performance of AVA 4.3C has also been demonstrated in animal models of critical illness [14]. AVA 4.3C was unable to reproduce the reductions in TVD and PVD demonstrated by manual analysis after induction of haemorrhagic shock in an ovine model. The intraclass correlation between the AVA 3.2 and AVA 4.3C was poor for all metrics. It should be noted that images were captured at the conjunctiva (rather than sublingual mucosa), however the lack of reliability remains pertinent to this work [14]. The failure of AVA 4.3C to discriminate between health and pathophysiologic states known to be associated with microcirculatory alterations is concerning. Considering these findings, we suggest that the use of AVA 4.3C should be avoided in future studies until it is validated against gold standard methods.

In the suite of software produced by Microvision Medical Pty Ltd, AVA 3.2 remains the most appropriate version to analyse SDF clips. Unfortunately, the current version of the Microscan camera is incompatible with AVA 3.2 and hence SDF clips must be captured in AVA 4.3C. Furthermore, AVA 4.3C records clips at a resolution of 1280 × 960, whereas AVA 3.2 manages clips at a resolution 640 × 480. SDF clips captured in AVA 4.3C must therefore be resized before import into AVA 3.2 for analysis; however, it is unclear whether resizing the clips compromises the quality of the analysis. The current study aims to: (i) validate that clip suitability for analysis (based on the MIQS) is preserved after resizing; (ii) to assess the interobserver reliability of AVA 3.2 for analysis of resized clips and (iii) to examine the agreement between automated analysis in AVA 4.3C and semi-automated analysis in AVA 3.2 of resized clips.

## Methods

### Population

The University of Queensland's ethical research committee approved this cohort study (2021/HE001851). The study was conducted in full compliance with principles of the “Declaration of Helsinki”, Good Clinical Practice (GCP), the National Statement on Ethical Conduct in Human Research (NHMRC, 2007), the Australian Code for the Responsible Conduct of Research (2007) and within the laws and regulations Australia.

Participants were recruited from the University of Queensland, St Lucia campus over a period of 12 months between December 2021 and December 2022. Individuals were excluded from the study if pregnant, had chronic health conditions, current malignancy or mobility limitation. The demographics of age, sex, height, weight were recorded.

### Recording of sublingual SDF videomicroscopy clips

**Fig. 1:**
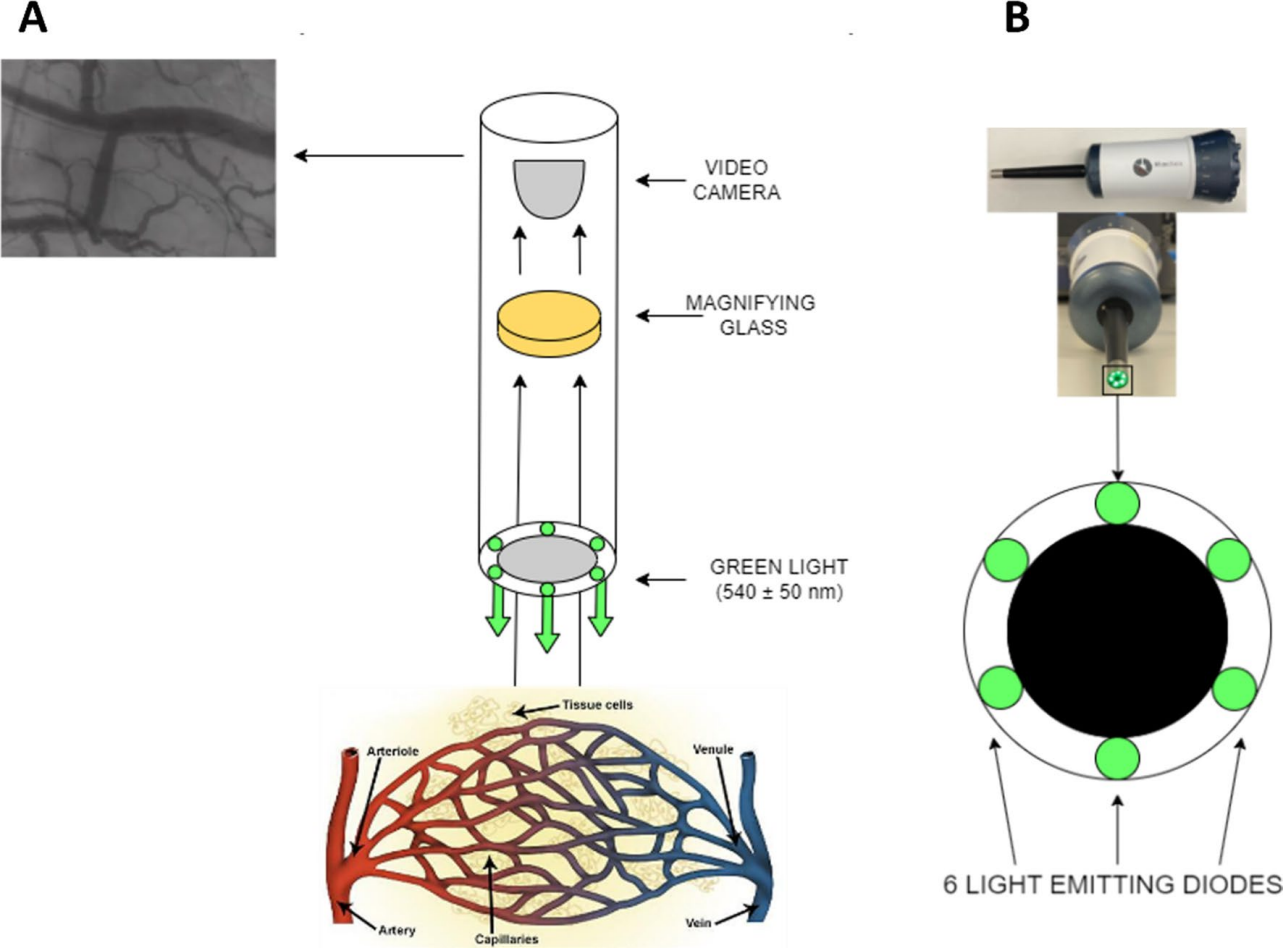
Microscan SDF camera schematic. Green light (540 nm) is emitted from a ring of light emitting diodes (LEDs) which is absorbed by haemoglobin. This allows visual differentiation of microcirculation from surrounding structures (**A**). The Microscan SDF videomicroscope used in the present study (**B**). Image adapted from [17]

Images of the sublingual microcirculation were obtained using SDF videomicroscopy (MicroScan, Micro Vision Medical, Amsterdam, the Netherlands) (Fig. 1). Participants were supine on a physiotherapy plinth with the backrest elevated to an angle of 40 degrees. Five video sequences of five second duration were recorded at the sublingual mucosa using AVA 4.3C (Automated Vascular Analysis (AVA) 4.3C, Academic Medical Centre, University of Amsterdam, Amsterdam, The Netherlands). Clips recorded using AVA 4.3C were saved as.avi files and coded for blinded analysis.

### Resizing of saved SDF clips

Saved.avi files recorded in AVA 4.3 were imported into ImageJ (ImageJ, US National Institutes of Health, Maryland, USA) and resolution was adjusted from 1280 × 960 to 640 × 480. Resized.avi files were saved and coded.

### Appraisal of image quality scores

All clips (before and after resizing) were assigned coded file names. A trained operator (RH) assigned each clip a MIQS. Briefly, the clip is assessed on multiple characteristics relating to the illumination, duration, focus, content, stability and pressure. A score of 0 (good), 1 (acceptable) or 10 (unacceptable) is assigned for each category. A clip is deemed acceptable for analysis if the sum of the scores is less than ten (i.e. a clip is not of sufficient quality for analysis if any of the categories are rated as unacceptable) [12].

### Analysis of SDF clips in AVA 3.2

All resized clips were analysed manually by two trained operators (RH and RL) in AVA 3.2 (Automated Vascular Analysis (AVA) 3.2, Academic Medical Centre, University of Amsterdam, Amsterdam, The Netherlands). The following parameters were calculated in accordance with consensus protocols for analysis of microcirculatory data: total vessel density (TVD), perfused vessel density (PVD), proportion of perfused vessels (PPV), microvascular flow index (MFI) and heterogeneity index (HI) [11].

The analysis protocol is outlined briefly here, however the reader is directed to published reference articles for further detail [11]. Clips are imported and stabilised then vessels are traced manually. Each vessel is then assigned a flow rating: 0 = absent (no flow), 1 = intermittent flow (absence of flow for at least 50% of the time), 2 = sluggish flow and 3 = continuous flow. A 3 × 3 grid is then superimposed over the stabilised clip. The TVD is calculated as the number of traced vessels crossing the gridlines relative to the length of the gridline. The PPV is the percentage of the total traced vessels that have a flow rating of 2 or 3. The PVD is then calculated as the product of TVD and PPV. The clip is then divided into four quadrants and overall flow is each quadrant is rated on the ordinal scale described above. The MFI is defined as the average flow rating across the four quadrants. After analysis of three clips for each subject, the HI is calculated as the difference between extreme values of PPV divided by the mean value [11].

### Analysis of SDF clips in AVA 4.3C

Clips were analysed in AVA 4.3C prior to resizing (at original resolution) using the ‘Perform Offline Analysis’ function. Analysis was performed automatically using the software, and the consensus perfusion and density data of the small vessels were recorded. AVA 4.3C does not report the HI or MFI.

### Statistical analysis

All statistical analyses were undertaken in Graphpad Prism 9.4.1. Data were assessed for normality by the Shapiro–Wilk test and visual inspection of q-q plots. MIQS scores before and after image resizing were compared using a Mann–Whitney U test (non-parametric). Agreement between two observers and between AVA 3.2 and AVA 4.3C were examined by Bland–Altman analysis. Bias and limits of agreement were reported for Bland–Altman analysis. The bias is the average difference between observers or analytical methods for a particular measure, and the limits of agreement are the 95% confidence intervals for the reported bias [15]. Regression lines were fitted to the Bland–Altman plots to determine whether bias between observers (or software versions) was proportional to the magnitude of the measured parameter [16]. Significance was set at *p* < 0.05.

## Results

### Study participants

Microcirculatory clips were captured in 14 adult females. 42 clips were therefore analysed by each observer. The mean and standard deviation for age and weight of enrolled participants was 53.3 (23.7) years and 65.9 (15.6) kilograms, respectively. Enrolment of exclusively female participants was to reduce the potential effect of sex-related heterogeneity in microcirculatory parameters.

### Preservation of image quality after resizing by MIQS

**Fig. 2:**
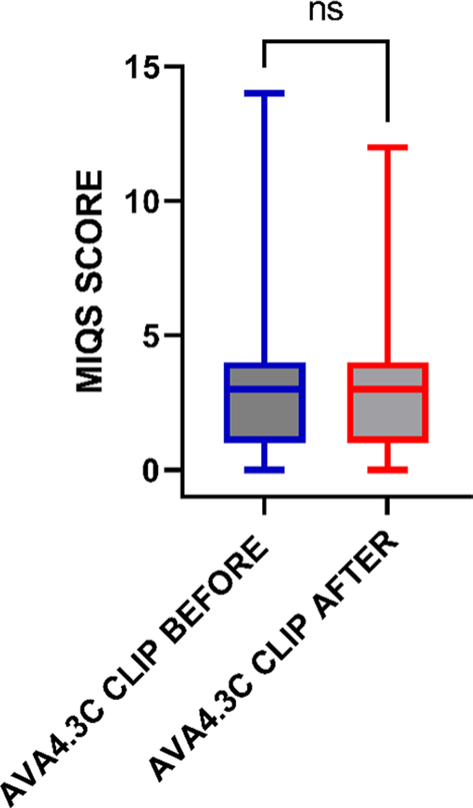
Effect of image resizing on MIQS. MIQS before (blue) and after (red) image resizing were compared by Mann–Whitney U test. Data presented as median and IQR

Table 1 presents the median and interquartile range (IQR) of the MIQS scores obtained by a single observer (RH) for the resized AVA 4.3C clips. There was no significant difference between MIQS before and after image resizing (Fig. 2).

**Table 1:** MIQS before and after image resizing

	MIQS		
	Before resizing	After resizing	*p*
MIQS	3 (1–4)	3 (1–4)	0.52

MIQS were calculated by a single observer and compared using a Mann–Whitney U test. Data presented as median (IQR)

### Interobserver reliability of semi-automated analysis of resized clips in AVA 3.2

All microcirculatory parameters, except for PPV, were normally distributed. Logarithmic transformation of PPV data failed to restore normality, hence limits of agreement and regression statistics for this metric must be interpreted cautiously.

**Fig. 3:**
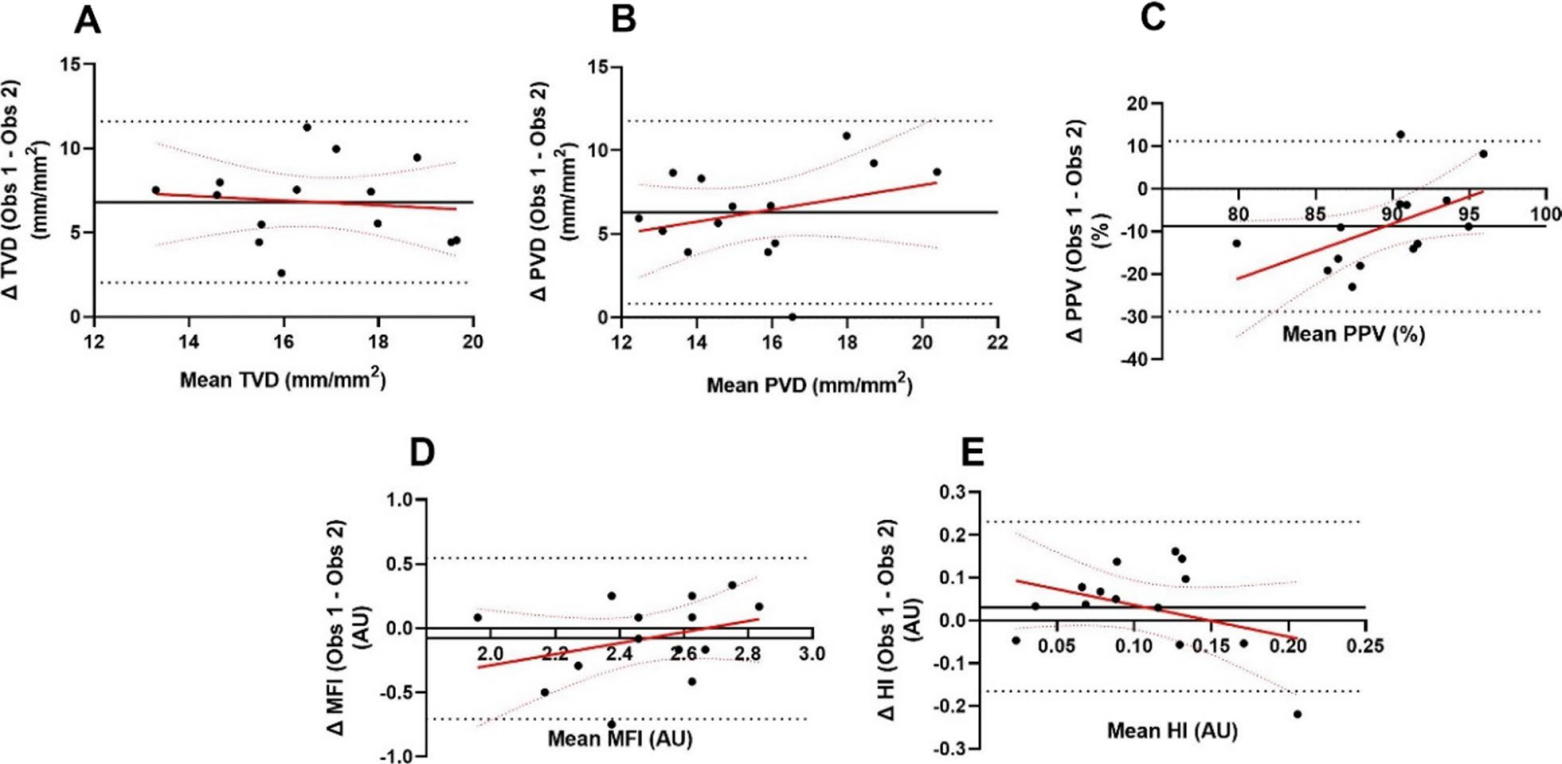
Bland–Altman plots for analysis of resized clips in AVA 3.2 by two trained observers. **A** Total vessel density; **B** perfused vessel density; **C** proportion of perfused vessels; **D** heterogeneity index; **E** microvascular flow index. Limits of agreement are shown by the black dotted lines, and bias between observers is shown by black solid lines. Regression and confidence intervals are shown in red solid and dotted lines respectively

Bland–Altman plots were generated to evaluate the interobserver agreement between two observers (RH and RL) in manual analysis of resized clips in AVA 3.2 (Fig. 3). A systematic positive bias for TVD, PVD and HI was observed, while a negative bias was observed for PPV and MFI (Table 2). Linear regression analysis of the Bland–Altman plots demonstrated consistent differences between the two observers across all microcirculatory parameters (Table 3). Linear regression coefficients for all parameters were non-significant demonstrating no proportional biases between observers (i.e. the magnitude of difference between observers for a parameter was not related to the mean value of that parameter) (Table 3).

**Table 2:** Bland–Altman statistics for interobserver reliability of AVA 3.2 clip analysis

	Lower LOA	Upper LOA	Bias
TVD (mm/mm^2^)	2.03	11.59	6.8
PVD (mm/mm^2^)	0.84	11.77	6.3
PPV (%)	− 28.77	11.18	− 8.79
MFI (AU)	− 0.71	0.55	− 0.08
HI (AU)	− 0.17	0.23	0.03

Bland–Altman limits of agreement and bias for semi-automated analysis of resized clips in AVA 3.2

**Table 3:** Regression statistics for interobserver reliability Bland–Altman plots

	Regression coefficient	R^2^	*p*
TVD (mm/mm^2^)	− 0.14 (− 0.93, 0.65)	0.01	0.70
PVD (mm/mm^2^)	0.36 (− 0.37, 1.1)	0.09	0.30
PPV (%)	1.28 (− 0.01, 2.6)	0.27	0.052
MFI (AU)	0.43 (− 0.37, 1.2)	0.10	0.26
HI (AU)	− 0.74 (− 1.9, 0.43)	0.13	0.20

Regression slope presented as coefficient (95% confidence interval)

### Comparison of microcirculatory parameters generated by AVA 3.2 and AVA 4.3C for resized clips

**Fig. 4:**
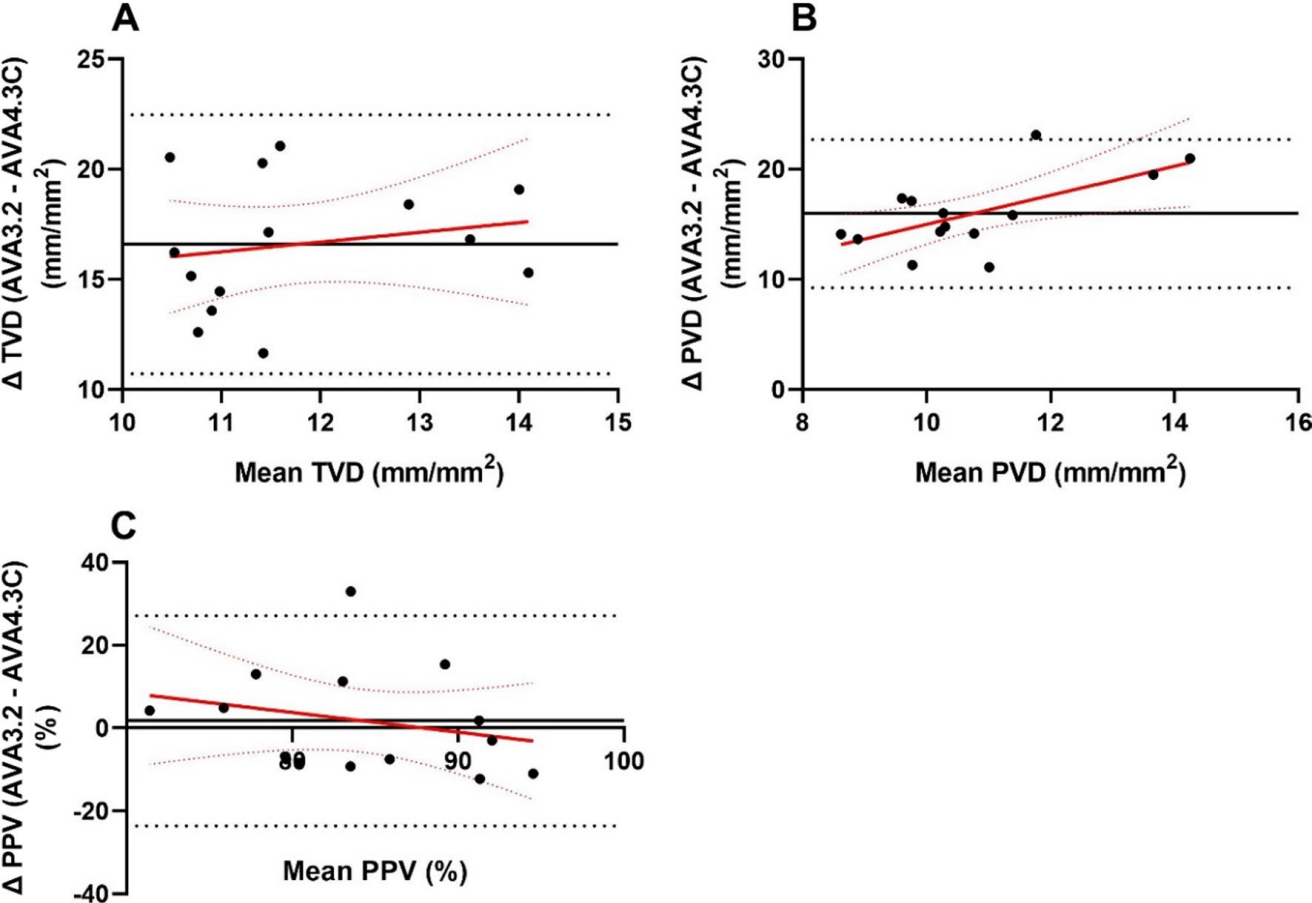
Bland–Altman plots for manual analysis (AVA 3.2) and automated analysis (AVA 4.3) of resized clips. **A** Total vessel density; **B** perfused vessel density; **C** proportion of perfused vessels. Limits of agreement are shown by the black dotted lines, and bias between observers is shown by black solid lines. Regression and confidence intervals are shown in red solid and dotted lines, respectively. Heterogeneity index (HI) and microvascular flow index (MFI) are not presented as these parameters are not provided in automated analysis by AVA 4.3C

Next, a comparison between microcirculatory parameters obtained by semi-automated analysis in AVA 3.2 (undertaken by RH) and automated analysis in AVA 4.3C. Bland–Altman plots were generated, revealing a marked positive bias for TVD, PVD and PPV (Fig. 4, Table 4). Regression analysis demonstrated a proportional bias for PVD (positive). Regression coefficients were non-significant for TVD and PPV, indicating that bias was not related to the mean values between analysis approaches (Table 5).

**Table 4:** Bland–Altman limits of agreement and bias for comparison of AVA 3.2 (manual) and AVA 4.3C (automated) analysis clips

	Lower LOA	Upper LOA	Bias
TVD (mm/mm^2^)	10.7	22.5	16.6
PVD (mm/mm^2^)	9.2	22.7	16.0
PPV (%)	− 23.6	27.1	1.8

Heterogeneity index (HI) and microvascular flow index (MFI) are not presented as these parameters are not provided in automated analysis by AVA 4.3C

**Table 5:** Regression statistics for AVA 3.2 compared to AVA 4.3C Bland–Altman plots

	Regression coefficient	R^2^	*p*
TVD (mm/mm^2^)	0.65 (− 0.99, 1.9)	0.04	0.51
PVD (mm/mm^2^)	0.48 (0.28, 2.4)	0.39	0.02
PPV (%)	− 0.47 (− 1.62, 0.68)	0.06	0.38

Regression slope presented as coefficient (95% confidence interval)

## Discussion

Microcirculatory monitoring has not yet been clinically adopted despite more than a decade of research and technological advances. Offline manual analysis is time consuming and occurs away from the patient. To expedite translation into the intensive care unit, several corporate entities have attempted to develop automated analysis software. Studies examining microcirculatory function in critically ill cohorts using automated analysis have reported contradictory findings to the wider literature [18]. The potentially confounding effect of unreliable automated analysis is a significant limitation of such studies.

Examination of microcirculatory function in various pathologic states is an active area of research. Until recently, analysis of microcirculatory clips was undertaken using AVA 3.2, which employs a semi-automated approach involving manual tracing of vessels and designating a semi-quantitative flow metric to each vessel. To improve time efficiency, more recent versions of the software (AVA 4.3C) now undertake vessel segmentation and flow analysis automatically. Poor reliability of automated analysis (AVA4x) has been demonstrated in several contexts [13, 14]. Presently, therefore, automated analysis using versions of AVA 4 is inappropriate for research use and clips must continue to be analysed in AVA 3.2 [13].

Further complicating SDF clip analysis is that the current generation of Microscan cameras record in a different resolution (1280 × 960) to that which is compatible with analysis in AVA 3.2 (640 × 480). We have therefore described an approach whereby clips are recorded using current generation cameras (as older generation cameras are no longer available), resized in ImageJ and imported into AVA 3.2 for manual analysis. In this study we have examined the effect of resizing clips on MIQS, and, the interobserver reliability of this process and the data generated from analysis. Finally, we examined agreement between AVA 4.3C and AVA 3.2 in analysing resized clips.

Current guidelines for microcirculatory assessment outline several image characteristics that impact analysis result but are unrelated to the underlying physiology of the patient. These include illumination, duration, focus, content, stability, and pressure. Errors in any of these parameters compromise the validity of the image for analysis. The MIQS quantifies these parameters to confirm that clips are appropriate for analysis; clips failing at this stage are discarded. Clips captured using AVA 4.3C were resized in ImageJ for analysis in AVA 3.2. We found that reducing the resolution of the clip did not significantly change the MIQS assigned by an experienced operator. This has not been previously demonstrated in the literature, however is an expected finding when considering the domains examined by the MIQS.

The MIQS score has been employed in the current study to confirm that changing resolution does not compromise suitability for analysis (mainly illumination and focus), not as an indicator of change in the analysis readout—MIQS scores were assigned before any analysis. Though it is unlikely resizing in ImageJ would change the MIQS, it is important that resized clips are evaluated before manual analysis to confirm MIQS scores are of sufficient quality. Indeed, we found that resizing the clips in ImageJ does not impact the MIQS score.

Resized clips were then imported into AVA 3.2 for semi-automated analysis by two experienced operators. A positive bias between observers was demonstrated for TVD, PVD and HI; while a negative bias was demonstrated for PPV and MFI. Regression analyses for each parameter were non-significant, indicating that bias between observers was not proportional to mean value of the parameter. The magnitude of the biases (difference between observers) is problematic when considered in the context of previous work in critically ill cohorts. For example, in the MICROSHOCK study [19], the difference in PVD between trauma patients who did and did not develop organ dysfunction after injury was approximately 2.6, whereas bias between observers in our study was approximately 6.3. Based on these data, analyses undertaken by separate observers should be compared acknowledging that biases between observers are likely greater than the clinically significant difference in parameters. Analysis for a particular study should therefore be undertaken by a single investigator. Though interobserver reliability for AVA 3.2 has not been previously examined, Scheuzger et al. [20] demonstrated good interrater reliability for manual analysis using CytoCam Tools. Cytocam Tools is produced by a different manufacturer, however, generates similar microcirculatory parameters to AVA 3.2. It should also be noted that Cytocam Tools is used to analyse incident darkfield (IDF) images (rather than SDF as used here) which may impact analysis reliability.

Finally, we examined the agreement between manual analysis (AVA 3.2) of resized clips and the automated analysis (AVA 4.3C) of the original clip. Significant proportional bias was observed for PVD and PPV between the two analysis methods. Poor agreement between automated and manual analysis has been previously reported [13].

We have demonstrated poor interobserver reliability between automated analysis in AVA 4.3C and manual analysis in AVA 3.2. Whether the differences between the two approaches is clinically significant remains to be seen. It is conceivable that automated analysis may be of clinical utility if it is able to effectively detect a change in microcirculatory function over time, even if the absolute values calculated deviate significantly from gold standard manual analysis. Assuming manual analysis has reasonable intraobserver reliability (as previously reported) [21], reliable detection of change in microcirculatory function by automated systems would require a consistent difference between automated and manual analysis for high and low values of microcirculatory parameters. We have demonstrated in this work that the magnitude of difference between automated and manual analysis changes with the value of the microcirculatory parameter. Specifically, for PVD a significant positive regression coefficient was evident on Bland–Altman analysis. In practice this suggests that for lower values of PVD analysis techniques had greater agreement than at higher values of PVD. The change in magnitude of deviation from the gold standard manual analysis across a spectrum of PVD values suggests that automated analysis would be unreliable in detecting deterioration of microcirculatory function in a patient.

The ability of automated analysis to detect changes over time has not been formally analysed and represents an important future direction from this work. The existing literature is variable with some studies reporting significant improvements in microcirculatory function with time [18] while others suggest that automated analysis by AVA4.1 (an earlier iteration of AVA4.3C) is insensitive to changes in microcirculatory function associated with a changing clinical state [13]. A relevant future study would involve serial imaging of the sublingual microcirculation in the intensive care unit with analysis of clips by automated software and manual techniques. A comparison could then be made between automated and manual analysis for reliability in detecting changes in microcirculatory function over time and predicting clinical outcome.

Current generation cameras that record in higher resolution produce images that improve the visible contrast between red blood cells in the microcirculation and surrounding stroma. The problem, as identified quantitatively in the present study, is not that higher resolution cameras are unreliable but that the automated analysis software that is coupled with higher resolution systems is unreliable. Manual analysis of microcirculatory clips therefore remains the gold standard. This is evidenced in the current guidelines for microcirculatory assessment [11] and supported by a dearth of high-quality evidence for a shift to automated analysis. Given that manual analysis requires lower resolution clips (software compatibility) than are recorded by current generation cameras, and, that older generation cameras are no longer available; it appears that the only option is to reduce the resolution of clips for manual analysis as reported here.

Until reliable automated analysis for current recording parameters is available or manual analysis software is updated for compatibility with higher resolution clips, increasing the resolution of cameras offers little benefit to clinical monitoring of the microcirculation. It does, however, appear that reliable automated analysis is approaching successful implementation. Once adopted more broadly, these higher resolution cameras will be of use. Our recommendation, based on the current study and the landscape of automated analysis approaches, is that clips be recorded using current generation technology in preparation for future automated analysis but continue to be manually analysed (using methods reported here) until automated protocols are validated.

Given the issues around reliability of current automated analysis, alternative analysis approaches are required that provide both reproducible and timely results. Microtools is a validated computer vision algorithm developed by Hilty et al. [22] for microcirculatory clip analysis. Microtools has been validated against semi-automated analysis in clinical populations and animal models [22, 23]. Currently, Microtools has been validated using IDF videomicroscopy clips. However, it represents a future avenue for analysis of SDF clips when validated. The developers of the AVA software have also recently released an updated version of the software, AVA 5, however this version remains to be validated (or reported) in the literature.

This current study has several key limitations. Firstly, we have examined clips from healthy volunteers. Though unlikely, it is possible that the current reliability data is not translatable to microcirculatory samples collected in pathologic states. Expansion of the current study population to include patients across a range of pathologies would therefore be of interest.

Enrolment of exclusively female participants in the current study may limit generalisation of reported microcirculatory parameters. Similarity in microcirculatory parameters between healthy males and females have, however, been previously reported [24]. The clips analysed in this study are derived from a database of human clips analysed in previous work comparing sublingual microcirculatory parameters in anaesthetised pigs to adult human [25]. This previous study was matched (female only) to reduce potential sex-related variation in microcirculatory parameters. Importantly, the purpose of this study was to examine interobserver reliability, rather than define normal ranges for microcirculatory parameters in healthy adults. We therefore suggest that the enrolment of exclusively female participants would have very limited (if any) effect on the interobserver reliability data reported here.

Though we have demonstrated interobserver reliability in this study; intraobserver reliability was not examined. High intraobserver reliability (> 0.8) for TVD, PVD and PPV has been demonstrated by repeated semi-automated analysis of SDF clips in AVA 3.0 (an earlier version of AVA 3.2 that involves a similar semi-automated analysis process) [21]. Given the similarities between these generations of AVA software, we expect the findings of Peterson et al. [21] to be generalisable to this work. Nonetheless, the current study would benefit from repeat analysis of our suite of resized clips. Finally, both observers in the current study were of similar experience levels. Examination of reliability between experienced and novice observers would be of interest with relevance to clinical utility of these tools.

## Conclusions

We have described an approach for semi-automated analysis of SDF clips captured in AVA 4.3C and examined interrater reliability. Though differences between observers were evident for microcirculatory parameters, these biases were consistent across the expected ranges for each parameter. These findings indicate that comparison of absolute values generated by different operators must be undertaken cautiously, which significantly limits the clinical utility of semi-automated analysis. This, and the poor reliability of fully automated analysis in AVA 4.3C represent significant hurdles for translation of microcirculatory analysis in the intensive care unit. At present, semi-automated analysis is suitable for research purposes (where generation of results is not time sensitive). In the scenario where microcirculatory assessment becomes part of critical care management, AVA 4.3C (though time efficient) is not fit for purpose.

## Data Availability

The datasets used and/or analysed during the current study are available from the corresponding author on reasonable request.

## Abbreviations

HVM: Handheld videomicroscopy
SDF: Sidestream darkfield
LEDs: Light emitting diodes
IDF: Incident darkfield illumination
TVD: Total vessel density
PVD: Perfused vessel density
PPV: Proportion of perfused vessels
MFI: Microvascular flow index
HI: Heterogeneity index
MIQS: Microcirculatory image quality score
AVA: Automated vascular analysis (software)
GCP: Good clinical practice
IQR: Interquartile range
